# The impact of exercise on tolerability of systemic treatment in metastatic colorectal cancer: A literature review

**DOI:** 10.1007/s11764-026-02002-8

**Published:** 2026-03-18

**Authors:** Laurien M. Buffart, Calvin G. Brouwer, Marlou-Floor Kenkhuis, Elske C. Gootjes, Henk M. W. Verheul, Tineke E. Buffart

**Affiliations:** 1 Department of Medical Biosciences, Radboud University Medical Center, Nijmegen, the Netherlands; 2 Department of Medical Oncology, Radboud University Medical Center, Nijmegen, the Netherlands; 3 Department of Medical Oncology, Erasmus Medical Center Cancer Institute, Rotterdam, the Netherlands; 4 Department of Medical Oncology, Amsterdam University Medical Center, Amsterdam, the Netherlands; 5 Department of Medical BioSciences, Radboudumc, Geert Grooteplein Zuid 10, Nijmegen, 6525 GA The Netherlands

**Keywords:** Metastatic colorectal cancer, Exercise, Systemic treatment, Toxicity, Physiology

## Abstract

**Abstract:**

Over half of patients with colorectal cancer (CRC) develop distant metastases and are often treated with multiple lines of systemic treatments, which can lead to severe toxicities. At least 40% of patients experience toxicity-induced treatment modifications in the first 3 months, which may impact anti-tumor effects. Exercise has the potential to limit toxicity and thereby prevent treatment modifications. This review discusses the role of physical exercise in limiting treatment toxicity in patients with metastatic CRC (mCRC). First an overview of the common toxicities of systemic treatment is presented. Second, the results from 15 observational studies examining associations of physical activity, fitness, and function with (toxicity-induced) treatment modification and survival outcomes are discussed, followed by the results from 8 exercise intervention studies in patients with mCRC. Finally, potential mechanisms of action by which exercise may impact toxicity of systemic treatments are described. Results highlight the potential benefits of exercise during systemic treatment. Although the number of studies is limited, observational studies found positive associations between physical activity, fitness, and function, and survival. Additionally, intervention studies revealed that exercise during systemic treatment is feasible and beneficial for patient-reported outcomes and physical fitness. Proposed mechanistic pathways by which exercise can limit toxicities including neurotoxicity, hematological toxicity, gastrointestinal toxicity, and fatigue are discussed. The potential of exercise on dermatological and immune-related toxicity of targeted and immunotherapy has yet to be investigated. Findings of this review emphasize the clear potential of exercise as an integral part of mCRC treatment, which should be confirmed in future trials.

**Implications for Cancer Survivors:**

Incorporating physical exercise during systemic treatment for metastatic colorectal cancer has the potential to help reduce or even prevent toxicity-induced treatment modifications, which could benefit survival outcomes.

## Introduction

Colorectal cancer (CRC) is one of the leading causes of cancer deaths worldwide. More than half of the patients diagnosed with CRC either have distant metastases at diagnosis or develop distant metastases during the disease course [1]. Treatment options vary by the location and extent of the metastases and may include local and systemic treatments.

Patients with multi-organ metastases of CRC are generally treated with multiple lines of systemic therapy. These treatments mostly include a combination of the chemotherapeutic agents fluoropyrimidines (5-fluorouracil (5FU), oral capecitabine or S1), oxaliplatin and irinotecan, and the targeted agents anti-VEGF (bevacizumab) and anti-EGFR (panitumumab or cetuximab) in RAS/BRAF wild-type left-sided mCRC [2–5]. Patients with BRAF mutated metastatic CRC (mCRC) can be treated with the combination of encorafenib (BRAF-inhibitor) and cetuximab [3]. Treatment with checkpoint inhibitors has become available for patients with mismatch repair (MMR) deficient mCRC [2, 6], and several other targeted agents are increasingly used to treat patients with mCRC, either as part of standard care or in clinical trials. Examples are TRK inhibitors, multiple combinations of HER2 inhibitors, and KRAS^G12C^ inhibitors with or without an anti-EGFR antibody [7]. As a consequence of these advances in medical treatments, current median survival for patients with mCRC has increased up to 24–30 months depending on tumor sidedness and molecular subtype [8].

Despite all new developments in treatment, in first line, patients with mCRC are typically treated with a chemotherapy backbone of 5FU derivatives with oxaliplatin and/or irinotecan with or without anti-VEGF/anti-EGFR. Over 40% of patients with mCRC treated with CAPOX (capecitabine and oxaliplatin) or FOLFOX (5FU and oxaliplatin) require treatment modifications, including dose reduction, treatment delay, treatment switch, or discontinuation, within the first three months of treatment, and up to 30% are admitted to the hospital due to chemotherapy-related toxicity [9]. Triplet chemotherapy (5FU/oxaliplatin/irinotecan) for treatment of mCRC increases treatment responses but is also associated with higher toxicity burden, with 50% experiencing ≥ grade 3 neutropenia (including 6% febrile neutropenia), and nearly 20% experiencing severe (≥ grade 3) diarrhea [5]. Toxicity-induced treatment modifications and hospitalization may reduce the benefit of treatment. Hence, limiting toxicity-induced treatment modification and hospitalization is important to optimize treatment benefit, daily functioning, and quality of life (QoL).

Observational studies in patients with cancer, including CRC, have shown that higher levels of physical activity, muscle mass and strength, and cardiorespiratory fitness are associated with better survival, less dose-limiting toxicity and higher odds of achieving 85% of relative dose intensity (RDI) of chemotherapy treatment [10–12]. Evidence from randomized controlled trials (RCT) showed exercise to be a powerful intervention to improve cardiorespiratory fitness and muscle strength [13]. Recently, the CHALLENGE trial showed that structured exercise after completion of adjuvant chemotherapy improves disease-free and overall survival in patients with colon cancer [14]. It is not clear to what extent exercise effects on colon cancer outcomes in this setting can be translated to other situations (e.g., exercising during cancer treatment) and to mCRC. Additionally, the underlying biological mechanisms of action are not fully understood. This knowledge is essential to understand the potential and limitations of exercise as an integral part of cancer treatment.

Pre-clinical studies in mice have shown that exercise can directly reduce tumor growth [15]. Hypothesized mechanisms include improved activation and infiltration of immune cells, normalized tumor vasculature, reducing hypoxia and increasing accessibility for systemic treatments, and alterations in intratumoral metabolic pathways [16]. Moreover, exercise can change the gut microbiota composition by increasing butyrate-producing bacteria, thereby inhibiting tumor growth [17].

Alternatively, exercise can improve cancer outcome indirectly via improved treatment tolerability resulting from enhanced coping and resilience [18] and reduced toxicity, thereby preventing treatment modifications and hospitalization. Few studies have reported on the effect of exercise on treatment relative dose intensity (RDI) in patients with CRC receiving adjuvant chemotherapy [19–22]. Three pilot RCTs with sample sizes of 23–39 patients with CRC found promising leads that RDI [19, 20] or the proportion of patients completing full dose [22] was higher in the combined aerobic and resistance exercise group compared to the control group. However, results from the FORCE trial among 181 patients with stage II and III CRC with RDI as primary endpoint did not show significant differences between the home-based resistance exercise group and the control group [21]. While the role of exercise on RDI also remains uncertain in patients receiving adjuvant chemotherapy, the scope of this review is to discuss its role in the management of treatment toxicity in patients with mCRC. First, we provide an overview of the common toxicities of systemic treatment used for patients with mCRC. Second, we summarize results from observational studies examining associations of physical activity, fitness and function with treatment toxicity and clinical outcome, as well as evidence from intervention studies examining the effects of exercise interventions in patients with mCRC. Third, we describe potential mechanisms of action by which exercise may prevent or reduce toxicity of systemic treatments in patients with mCRC.

## Common toxicities of systemic treatments in mCRC

In the complex decision making for palliative treatments, the anti-tumor effect is balanced with potential toxicity to aim for clinical benefit with maintained or improved QoL. Common toxicities of combination chemotherapy are myelosuppression (i.e., neutropenia, thrombocytopenia, anemia), gastrointestinal toxicity (e.g., nausea, diarrhea), neuropathy, hand-foot syndrome, alopecia, and fatigue [5, 8, 9]. Targeted agents have substantially different toxicity profiles compared to chemotherapy. Anti-EGFR is often associated with dermatological toxicities (e.g., rash, acneiform dermatitis), and checkpoint inhibitors can induce immune-related adverse events such as thyroiditis, hepatitis, colitis, pneumonitis, and hypophysitis. Additionally, fatigue is a common side effect of basically all anti-cancer treatments. Table 1 presents the most common toxicities of systemic treatments generally used for patients with mCRC. Often, only grade 3–4 toxicity is reported, while the proportion of patients with any grade toxicity is much higher than those experiencing toxicity of grade ≥ 3 (Table 1). A previous study showed that, on average, patients with mCRC experience at least 5 toxicities of any grade during 3 months of systemic treatment, and that multiple concurrent low-grade adverse events can have a cumulative impact on self-reported physical function [9]. Consequently, even small reductions in toxicity, including the low-grade toxicity, can have important clinical implications.

**Table 1:** Overview of toxicities associated with systemic treatment of mCRC

Study/Toxicity (%)	TRIBE 2 [5]				ASPECCT [4]				BEACON CRC [3]				Keynote 177 [2]				Checkmate 142 [6]	
Type of treatment	FOLFOX-B		FOLFOXIRI-B		Panitumumab		Cetuximab		Encorafenib + Cetuximab		Irinotecan/FOLFIRI + Cetuximab		Pembrolizumab		FOLFOX-B/C or FOLFIRI-B/C		Ipilimumab/Nivolumab	
Sample size (n)	336		336		496		350		216		193		153		143		45	
Toxicity grade	Any	3 +	Any	3 +	Any	3 +	Any	3 +	Any	3 +	Any	3 +	Any	3 +	Any	3 +	Any	3 +
*Neurotoxicity*																		
Neuropathy	59%	1%	49%	2%	NA	NA	NA	NA	NA	NA	NA	NA	2%	0%	22%	2%	NA	NA
*Hematological toxicity*																		
Hemoglobin	44%	2%	58%	3%	6%	2%	6%	3%	32%	4%	44%	4%	18%	5%	22%	10%	NA	NA
Thrombocytopenia	27%	2%	27%	2%	NA	NA	NA	NA	NA	NA	NA	NA	NA	NA	NA	NA	NA	NA
Neutropenia	50%	21%	73%	50%	NA	NA	NA	NA	NA	NA	NA	NA	2%	0%	21%	15%	NA	NA
Febrile neutropenia	NA	3%	NA	6%	NA	NA	NA	NA	NA	NA	NA	NA	NA	NA	NA	NA	NA	NA
*Gastrointestinal toxicity*																		
Diarrhea	41%	6%	67%	17%	18%	2%	18%	2%	33%	2%	48%	10%	44%	6%	62%	11%	16%	0%
Nausea	45%	3%	55%	6%	14%	< 1%	11%	1%	34%	< 1%	41%	1%	31%	3%	59%	4%	13%	0%
Vomiting	18%	1%	32%	3%	12%	2%	10%	1%	21%	1%	29%	3%	22%	1%	37%	5%	NA	NA
Constipation	NA	NA	NA	NA	8%	< 1%	14%	< 1%	15%	0%	18%	1%	17%	1%	31%	1%	NA	NA
Decrease appetite	NA	NA	NA	NA	14%	1%	16%	1%	27%	1%	27%	3%	24%	0%	41%	5%	NA	NA
Weight loss/anorexia	14%	2%	22%	1%	5%	< 1%	4%	0%	10%	< 1%	6%	0%	5%	1%	12%	1%	NA	NA
Stomatitis	34%	3%	45%	5%	6%	< 1%	7%	0%	6%	0%	23%	2%	7%	0%	30%	4%	NA	NA
Abdominal pain	NA	NA	NA	NA	12%	3%	17%	3%	23%	2%	25%	5%	24%	5%	29%	6%	NA	NA
Mucositis	NA	NA	NA	NA	4%	< 1%	5%	< 1%	NA	NA	NA	NA	5%	0%	19%	1%	NA	NA
*Fatigue*																		
Fatigue	NA	NA	NA	NA	15%	3%	17%	4%	30%	4%	27%	4%	38%	4%	50%	9%	16%	0%
Asthenia	53%	6%	57%	7%	7%	1%	8%	1%	21%	3%	25%	5%	12%	2%	22%	4%	16%	2%
*Dermatological toxicity*																		
Skin toxicity (dermatitis acneiform)	NA	NA	NA	NA	27%	3%	27%	3%	29%	< 1%	39%	3%	1%	1%	1%	1%	47%	0%
Rash	NA	NA	NA	NA	50%	5%	52%	4%	12%	0%	14%	2%	13%	1%	11%	1%	16%	0%
Palmar-plantar erythrodysesthesia	8%	1%	5%	0%	NA	NA	NA	NA	4%	< 1%	7%	0%	12%	0%	17%	1%	NA	NA
Dry skin	NA	NA	NA	NA	17%	< 1%	16%	0%	11%	0%	7%	1%	12%	0%	9%	0%	NA	NA
Pruritus	NA	NA	NA	NA	17%	< 1%	17%	< 1%	9%	0%	5%	0%	16%	0%	8%	1%	36%	0%
*Immune-related toxicity*																		
Hypothyroidism	NA	NA	NA	NA	NA	NA	NA	NA	NA	NA	NA	NA	12%	0%	2%	0%	18%	2%
Colitis	NA	NA	NA	NA	NA	NA	NA	NA	NA	NA	NA	NA	10%	3%	0%	0%	20%	4%
Pneumonitis	NA	NA	NA	NA	NA	NA	NA	NA	NA	NA	NA	NA	45%	0%	1%	0%	4%	2%
Hepatitis	NA	NA	NA	NA	NA	NA	NA	NA	NA	NA	NA	NA	6%	3%	0%	0%	11%	4%
Adrenal insufficiency	NA	NA	NA	NA	NA	NA	NA	NA	NA	NA	NA	NA	4%	1%	0%	0%	NA	2%
*Other abnormal laboratory values*																		
ALAT	NA	NA	NA	NA	NA	NA	NA	NA	17%	0%	26%	3%	14%	3%	11%	2%	NA	2%
ASAT	NA	NA	NA	NA	NA	NA	NA	NA	14%	1%	20%	2%	16%	3%	8%	2%	NA	2%
Creatine kinase	NA	NA	NA	NA	NA	NA	NA	NA	3%	0%	7%	0%	NA	NA	NA	NA	NA	NA
Bilirubin	NA	NA	NA	NA	NA	NA	NA	NA	7%	2%	8%	3%	NA	NA	NA	NA	NA	NA
Potassium	NA	NA	NA	NA	8%	3%	5%	2%	NA	NA	NA	NA	8%	1%	17%	6%	NA	NA
Calcium	NA	NA	NA	NA	6%	2%	3%	1%	NA	NA	NA	NA	NA	NA	NA	NA	NA	NA
Magnesium	NA	NA	NA	NA	27%	7%	18%	3%	NA	NA	NA	NA	NA	NA	NA	NA	NA	NA
Creatinine	NA	NA	NA	NA	NA	NA	NA	NA	50%	2%	34%	1%	NA	NA	NA	NA	NA	2%
*Other toxicities*																		
Headache	NA	NA	NA	NA	3%	0%	7%	0%	19%	0%	3%	0%	14%	0%	15%	0%	NA	NA
Insomnia	NA	NA	NA	NA	5%	0%	9%	0%	11%	0%	6%	0%	NA	NA	NA	NA	NA	NA
Peripheral oedema	NA	NA	NA	NA	5%	1%	8%	1%	8%	0%	7%	1%	12%	0%	8%	1%	NA	NA
Arthralgia	NA	NA	NA	NA	NA	NA	NA	NA	19%	1%	1%	0%	18%	1%	5%	0%	20%	0%
Myalgia	NA	NA	NA	NA	NA	NA	NA	NA	13%	< 1%	2%	0%	NA	NA	NA	NA	NA	NA
Venous thrombosis	10%	6%	10%	4%	NA	NA	NA	NA	NA	NA	NA	NA	NA	NA	NA	NA	NA	NA
Hypertension	29%	10%	23%	7%	NA	NA	NA	NA	NA	NA	NA	NA	12%	7%	11%	5%	NA	NA
Infusion reaction	NA	NA	NA	NA	3%	< 1%	15%	2%	NA	NA	NA	NA	2%	0%	8%	1%	NA	NA
Pyrexia (fever)	12%	0%	16%	0%	6%	< 1%	11%	1%	16%	1%	14%	1%	18%	1%	14%	0%	11%	0%
Cough	NA	NA	NA	NA	8%	0%	8%	0%	7%	< 1%	5%	0%	17%	0%	16%	0%	NA	NA
Dyspnea/respiratory failure	NA	NA	NA	NA	4%	1%	8%	1%	11%	1%	9%	3%	14%	1%	10%	0%	NA	2%
RTI	NA	NA	NA	NA	3%	< 1%	6%	0%	NA	NA	NA	NA	10%	0%	6%	0%	NA	NA
UTI	NA	NA	NA	NA	3%	< 1%	6%	0%	NA	NA	NA	NA	9%	1%	11%	3%	NA	NA

LWI = ALAT, alanine aminotransferase; ASAT = Aspartate aminotransferase; RTI = Respiratory tract infection; UTI = urinary tract infection; NA = not assessed, Percentages are given as rounded numbers

## Physical activity, fitness, and function and mCRC treatment-related toxicity and outcome

Physical activity can be defined as any bodily movement via skeletal muscles that results in energy expenditure, whereas exercise is a sub-form of physical activity that is planned, structured, and repetitive and intended to improve physical fitness or health [23]. In contrast to physical activity being a behavior, physical fitness is a set of attributes that people have or achieve. Important components of physical fitness include cardiorespiratory fitness and muscle strength and endurance. Physical function is defined as the ability to perform basic movements (e.g., walking, stair climbing, standing up from a chair, lifting or carrying) or more complex daily life activities (e.g., self-care, home management, shopping, and getting to appointments) [24]. Exercise interventions can be used to improve physical fitness and physical function.

**Fig. 1:**
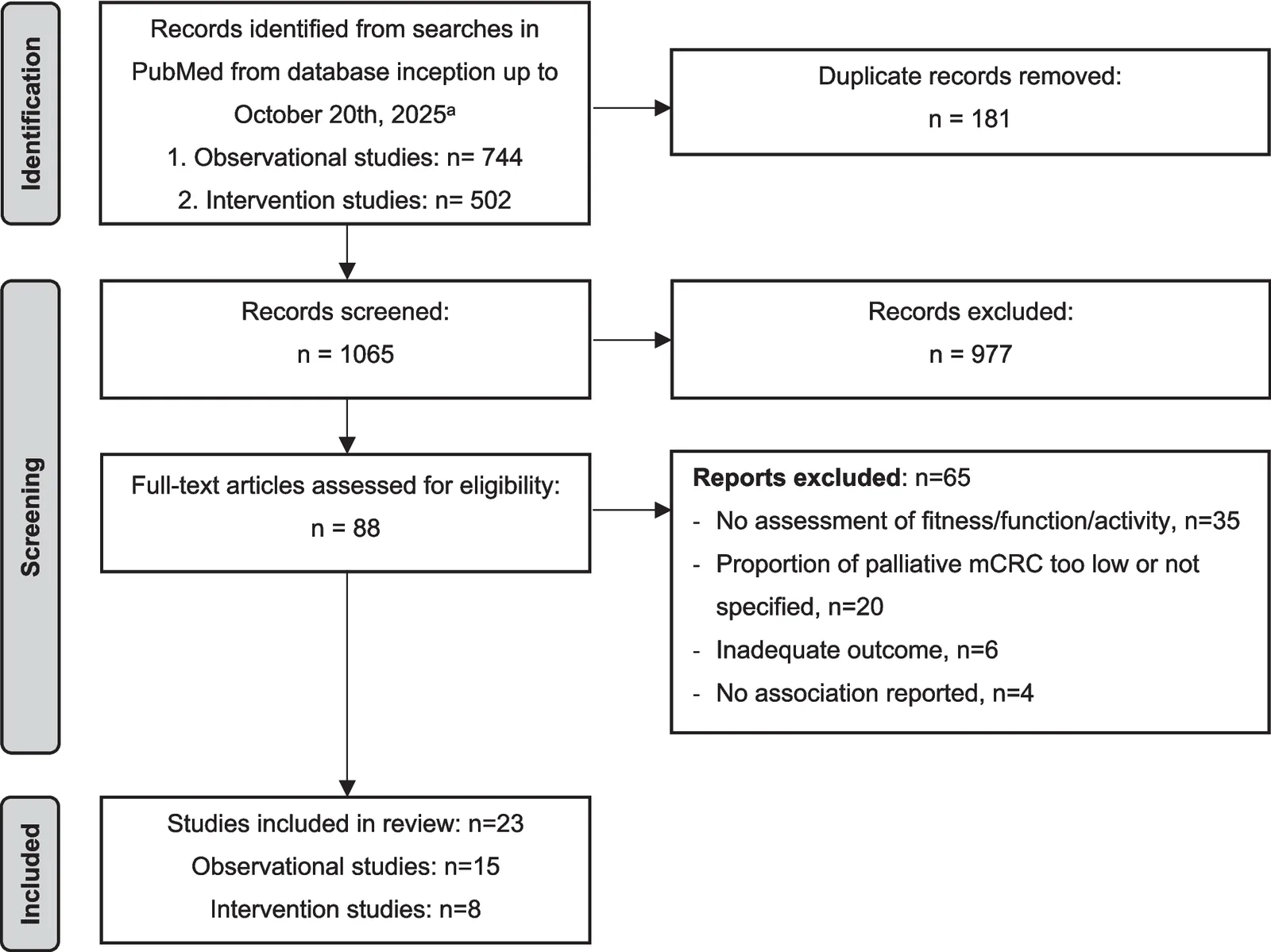
Flow chart of the study selection process ^a^Searches: 1. Observational studies examining the associations of physical activity, fitness, and function with (toxicity-induced) treatment modification and outcome in patients with mCRC. Key search terms: 1) colorectal cancer, 2) palliative or systemic treatments, 3) physical activity, fitness, function, exercise, sedentary behavior, and muscle strength, and 4) toxicity, progression, or survival. 2. Intervention studies examining the effect of exercise on toxicity and (patient-reported) outcomes in patients with mCRC. Key search terms: 1) colorectal cancer, 2) palliative or systemic treatments, 3) physical activity or exercise, and 4) trials. Exclusion of studies: that solely measured performance status with the ECOG/WHO or the Karnofsky scores included a heterogeneous group of patients with advanced cancer with less than 1/3 being diagnosed with mCRC or where the number of patients with mCRC was not specified

The potential of physical activity, fitness, and function for clinical outcome in patients with mCRC was examined using fifteen [10, 25–38] observational studies and eight [39–46] intervention studies, which were identified using systematic literature searches in PubMed covering all publications from database inception up to October 20th, 2025 (Fig. 1). We excluded studies focusing solely on clinician’s rated performance classifications to determine whether patients can tolerate treatment, such as the ECOG/WHO or Karnofsky performance status scores, as they are not valid proxy measures for physical activity, exercise, physical fitness, or physical functioning [47].

Six observational studies [10, 25–27, 29, 30] focused on physical activity, eight [31–38] focused on physical fitness or function, and one [28] assessed physical activity, fitness, and function (Table 2). Five of these studies focused on treatment-related toxicity [25, 27, 28, 31, 38]. Of three studies examining the association between physical activity (one with objective measures [25] and two with patient-reported outcomes [27, 28]) and the presence of toxicity ≥ grade 3, one study [27] found that the time to first ≥ grade 3 toxicity was significantly lower for patients reporting to engage in physical activity ≥ 9 METhrs/week compared to less active patients. All three studies examining the association between objectively assessed physical fitness or function and dose-limiting or grade ≥ 3 toxicity found no significant association [28, 31, 38].

**Table 2:** Overview of observational studies examining the association of physical activity, fitness, or function with treatment-related toxicity and outcome in patients with mCRC

First author (year of publication)	Study sample	Determinant	Outcome	Results
*Physical activity*				
*Objective measure*				
Innominato [25]	130 mCRC receiving first-line chemotherapy	Average wrist movements per minute over 72 h measured with a wrist-worn accelerometer	Grade 3–4 toxicity; tumor response; PFS; OS	- No significant associations with toxicity - No significant associations with survival
Piringer [26]	22 mCRC treated with regorafenib	Median steps/day assessed with a pedometer over one week	PFS	- No significant association with survival
*Patient-reported measure*				
Guercio [27]	1218 mCRC receiving first-line chemotherapy	Leisure time physical activity within one month after therapy initiation, expressed in METhr/wk	Grade 3–4 toxicity; PFS; OS	- Significantly lower PFS for < 3 METhrs/week vs ≥ 18 METhrs/wk (HR = 0.83, 95%CI = 0.70; 0.99) - No statistically significant association between PA and OS (HR = 0.85, 95%CI = 0.71; 1.02) - Significantly more toxicity for < 9 vs. ≥ 9 METhrs/wk (HR = 0.73, 95%CI = 0.62; 0.86)
Retornaz [28]	97 older patients with CRC of whom 62.9% mCRC	Canadian Study of Health and Aging Risk Factor Questionnaire, assessed at baseline	Grade 3–4 toxicity; OS	- No significant associations with toxicity - No significant association with survival
Smit [10]	293 mCRC receiving systemic treatment	SQUASH questionnaire, assessed within 60 days after diagnosis; METhrs/wk and MVPAhrs/wk in tertiles	OS	- Significantly lower mortality in middle (HR = 0.491, 95%CI = 0.299; 0.807) and highest (HR = 0.485, 95%CI = 0.303; 0.778) tertile of METhrs/wk - Significantly lower mortality in the highest tertile of MVPAhrs/wk compared to the lowest tertile (HR = 0.476, 95%CI = 0.278; 0.816)
Sun [29]	34 mCRC not on active treatment participating in a traditional Chinese herbal medicine trial	IPAQ-7 questionnaire, dichotomized into high (≥ 3000 MET-min/wk) vs. low (< 3000 MET-min/wk) activity	OS	- Significantly lower mortality in high active vs. low active (HR = 0.13, 95%CI = 0.03; 0.66)
Walter [30]	3121 CRC of whom 469 mCRC (presented separately)	Self-reported lifetime and latest leisure time physical activity	CRC-specific survival; RFS; DFS; OS,	- No significant association with survival
*Physical fitness & function*				
*Objective measure*				
Retornaz [28]	97 older patients with CRC of whom 62.9% mCRC	Grip strength, TUG, and one leg balance at baseline	Grade 3–4 toxicity; OS	- No significant associations with toxicity - No significant association with survival
Versteeg [38]	103 older patients receiving palliative chemotherapy, of whom 52% CRC	Grip strength	Dose-limiting toxicity; OS	- No significant association with toxicity - Higher grip strength (in kg) associated with longer OS (HR = 1.03, 95%CI = 1.00;1.05) - Patients with normal muscle strength had higher OS than patients with poor muscle strength (HR = 1.75, 95%CI = 1.02; 3.00)
Ward [31]	45 mCRC, receiving first-line chemotherapy	TUG dichotomized into good (≤ 10 s) vs. bad (> 10 s)	Grade 3–5 toxicity; OS	- No significant association with toxicity - Good TUG was significantly associated with lower mortality (HR = 0.31, 95%CI = 0.12; 0.78)
*Patient-reported measure*				
Comella [32]	310 mCRC receiving first-line chemotherapy	EORTC QLQ-C30 PF at baseline	Chemotherapy response rate; PFS; OS	- PF was significantly associated with OS (HR = 0.988, 95%CI = 0.981; 0.995) - PF was not significantly associated with chemotherapy response rate or PFS
Frank [33]	258 rectal cancer of whom 50 mCRC receiving first-line chemotherapy (presented separately)	EORTC QLQ-C30 PF at baseline	OS	- No significant association between PF and OS (HR = 0.70, 95%CI = 0.49; 1.01)
Grande [34]	100 palliative colorectal/lung cancer of whom 46 mCRC	EORTC QLQ-C30 PF and SF-36 PF and physical component summary	OS	- No significant associations for OS
Mol (2016)[35]	555 mCRC receiving first-line treatment	EORTC QLQ-C30 PF at baseline dichotomized into good (> 66.7%) vs poor (≤ 66.7%)	OS	- Good PF was significantly associated with lower mortality (HR = 0.57, 95%CI = 0.46; 0.72)
Ooki [36]	127 mCRC receiving first- line treatment plus cetuximab	EORTC QLQ-C30 PF at baseline	High (≥ 20%) vs. low (< 20%) early tumour shrinkage	- No statistically significant difference in PF of high (87.5 ± 2.2) vs low (80.4 ± 2.9) early tumour shrinkage, *p* = 0.051
Quinten [37]	1492 patients with CRC (from 3 trials) of whom 886 mCRC	EORTC QLQ-C30 PF at baseline	OS	- Higher PF was significantly associated with lower mortality (HR = 0.93, 95%CI = 0.96; 0.99)

Abbreviations: CI = confidence interval; CRC = colorectal cancer; DFS = disease-free survival; EORTC QLQ-C30 = European Organization for Research and Treatment Quality of Life Questionnaire Core 30; HR = hazard ratio; IPAQ = International Physical Activity Questionnaire; mCRC = metastatic colorectal cancer; MET = metabolic equivalent of task; MVPA = moderate to vigorous intensity physical activity; OS = overall survival; PA = physical activity; PF = physical function; PFS = progression free survival; RFS = recurrence-free survival; SF-36 = Short form 36; TUG = timed up and go test

Associations with survival outcomes were investigated in 14 studies [10, 25–35, 37, 38] and one study focused on early tumor shrinkage [36]. Among the five studies using self-reported measures of physical activity [10, 27–30], three [10, 27, 29] reported that higher physical activity was associated with better (progression-free) survival. Two studies with smaller sample sizes that used objective measures of physical activity found no significant associations with survival [25, 26]. Of the five studies using patient-reported measures of physical function [32–35, 37], three [32, 35, 37] reported that better physical functioning was associated with improved overall survival. Additionally, two [31, 38] studies using objective measures of physical fitness or function reported a positive association with overall survival, but one [28] did not.

The sample sizes of exercise intervention studies among patients with mCRC ranged between 21 and 101. Five studies focused on feasibility or safety as primary endpoints [39, 41, 42, 44, 45] and three other studies compared an exercise intervention group to a control group using a RCT design with mobility [40], chemotherapy-induced peripheral neuropathy (CIPN) [46], and fatigue and QoL [43] as primary endpoints (Table 3). The interventions differed in terms of delivery mode (supervised vs. home-based) and exercise type (aerobic vs. resistance exercises). Adherence to the exercise interventions varied from 47 to 88%. Additionally, significant beneficial effects of exercise were found for mobility [40], CIPN [46], and fatigue [43], and for QoL in the subgroup with good exercise adherence [43]. Although not consistent across studies, several beneficial effects of exercise were found on a variety of secondary endpoints including QoL [42, 45], psychological distress [45], sleep quality or duration [40, 42, 44], aerobic capacity [41, 42, 45], muscle mass [44] or strength [42, 46], pain [42], physical activity [42], RDI [41, 45], and time to start postoperative chemotherapy treatment [45] (Table 3).

**Table 3:** Overview of exercise intervention studies in patients with mCRC

First Author (year of publication)	Description of study sample	Study design	Exercise intervention	Adherence	Outcome measures	Results
Brouwer [39]	21 patients with mCRC receiving first line treatment participating in the exercise arms	Three arm RCT: AET + RET vs AET + HIIT vs control	Two 60-min supervised exercise sessions per week during six 3-week or eight 2-week cycles of treatment AET: continuous moderate intensity aerobic exercise Combined with: RET: resistance exercises of 6 muscle groups, 2 sets of 10 repetitions at 70–80% of 1RM HIIT: 25 min HIIT on a cycle ergometer	Median exercise attendance: 67% (IQR 35–91%) Median ExRDI: 61% (IQR 31–68%)	Primary: feasibility, patient satisfaction and perceived benefits (via qualitative interviews)	- Patient satisfaction: score 8 out of 10 (IQR 8–9) - Patients’ perceptions: • Patients valued the guidance and knowledge of the physical therapist and expressed interindividual preferences regarding training content • Patients’ experiences: improved physical and mental wellbeing and better treatment tolerance • Perceived exercise barriers: treatment toxicity, physical problems, and hospital appointments • Perceived exercise facilitators: adequate tailoring and internal or external motivation
Cheville [40]	66 metastatic lung/CRC of whom 32 mCRC. No treatment specified	Two-arm RCT: exercise vs. control	Eight-week incremental home-based pedometer-based walking (≥ 4x/week, 10% increase per 2 weeks) and resistance exercise (≥ 2x/week 4 sessions (2 upper and 2 lower body exercises), 10–15 repetitions per exercise	Proportion of patients participating in resistance exercise training 4 times/week and ≥ 10% increase in step counts every 4 weeks: 76.9%	Primary: self-reported mobility (AM-PAC) Secondary: pain (NRS); HRQoL (FACT-G); fatigue (FACT-F); sleep quality (NRS); activities of daily living (AM-PAC)	- Significant between-group differences in: • mobility (*p* = 0.01) • fatigue (*p* = 0.02) • sleep quality (*p* = 0.05) - No significant differences in activities of daily living, HRQoL and pain
Chiarotto [41]	35 metastatic cancer of whom 12 mCRC receiving palliative chemotherapy (presented separately)	Pre-post single-arm study compared with age- and sex-matched nonparticipants	Group-based supervised resistance (2 sets of 10 repetitions for 5–7 exercises), and aerobic exercise (walking/cycling up to 45 min) session for 75 min/wk, once a week as long as the patient wished to participate	Mean proportion of sessions attended: 73.1% (95%CI = 67.0; 79.4)	Primary: feasibility Secondary: RDI; OS; HRQoL (FACIT); fatigue (PFS); aerobic capacity (6MWT)	- Participation in the exercise trial was not significantly associated with OS - RDI was significantly higher in participants than in nonparticipants (96% vs. 86% *p* = 0.035) - No significant changes in QoL and fatigue - Significant improvement in aerobic capacity (*p* = 0.002)
Jensen [42]	26 metastatic GI cancer of whom 11 mCRC receiving palliative systemic treatment	Two-arm RCT: AET vs. RET	Twelve-week supervised exercise intervention, two 45-min sessions per week, RPE 13–15 RET: exercises of large muscle groups, 2–3 sets of 15–25 repetitions per exercise at 60–80% of 1RM AET: bicycle ergometer 10–30 min at 60–80% of predetermined pulse rate	Median proportion of session attended: RET: 72% (range: 25%—92%); AET: 59% (range: 4; 83%)	Primary: feasibility Secondary: HRQoL (EORTC-QLQ-C30) RET only: Muscle strength (indirect 1-RM) AET only: Cardiorespiratory fitness (PWC130); Physical activity (SenseWear®); Freiburger Questionnaire of Physical Activity); sleep (SenseWear®); Blood markers (blood cell counts and markers of chronic inflammation, glucose metabolism and lipid metabolism)	Both groups: - Significant improvement in fatigue (*p* = 0.003), global QoL (*p* = 0.008), role functioning (*p* = 0.04), sleep duration (*p* = 0.028) - No significant improvement in objectively assessed physical activity and blood markers RET: - Significant improvement in muscle strength (leg muscles: *p* = 0.001; biceps: *p* = 0.017; back: 0.048; knee flexors: *p* = 0.002) AET: - Significant improvement in self-reported physical activity (*p* = 0.034) - No significant improvement in cardiorespiratory fitness
Navigante [43]	101 advanced GI cancer, of whom 52 mCRC during first-line chemotherapy	Two-arm RCT: exercise vs. control	Programmed and directed physical exercise two times a week (1 in the clinic, 1 at home with a personal trainer) for 45 min for 12 weeks including aerobic and resistance exercises	47% of patients reached ≥ 50% of planned exercise in at least 50% of their weeks in the study	Primary: fatigue (NRS) and HRQoL (FACT-G) Secondary: grip strength (hand-held dynamometer), body composition (bioimpedance)	- Significant reduction in fatigue in the intervention group vs. the control group (*p* = 0.019) - No significant between-group differences in QoL, grip strength, and body composition
Park [44]	30 advanced GI cancer, of whom 11 mCRC undergoing palliative chemotherapy	Pre-post single-arm study	Aerobic and resistance exercise 30–40 min of low intensity (9–10 RPE; 30% HRR) progressing to moderate (RPE 11–13; 40–50% HRR) and high (RPE 14–15; 70% HRR) intensity for 6 weeks (5 days/week) plus additional 6 weeks when compliant	53% completed over 80% of exercise sessions. Median number of total exercise sessions: 24	Primary: feasibility Secondary: handgrip strength; SMI (APCT); 6MWT; body composition (bioimpedance, BMI); blood biomarkers (Hb, TLC, albumin, prealbumin, transferrin, CRP); QoL (EORTC QLQ-C30); PFS; OS	- Improvement in insomnia subscale (*p* = 0.042) - Reduction in SMI (*p* = 0.03), with larger reductions in poor compliant group (*p* = 0.03) and no significant change in compliant group (*p* = 0.34) - No significant changes in total lean mass, BMI, 6MWT, handgrip strength, blood biomarkers - The median PFS of good compliant group was numerically higher than the poor compliance group, but this difference was not statistically significant (7.6 months vs.2.2 months, HR = 0.636, 95%CI = 0.284;1.427, *p* = 0.272) - Good compliant group had longer OS than poor compliant group (HR = 0.202, 95%CI = 0.046;0.878, *p* = 0.033)
Thomsen [45]	52 patients with colorectal liver metastasis who received liver resection	Two-arm RCT: exercise vs control (randomized 2:1)	Unsupervised home-based 30–50 min low–high intensity cycling 5 times per week for 8 weeks, initiating the day after post-operative hospital discharge	Median session attendance rate: 93% (IQR 59%) of planned sessions Median exercise dose: 66% (IQR 72%)	Primary: number of SAEs Secondary: non-SAE and unscheduled hospitalizations (medical records), RDI of post-operative chemotherapy, patient-reported adverse events (PRO-CTCAE) cardiorespiratory fitness (peakVO_2_ assessed on cycle ergometer), physical performance (10 m gait speed, stair climbing power), body composition (DEXA), blood chemistry (e.g., CRP), HRQoL (FACT-G/C), psychological distress (HADS)	- SAE: 11 in 8 patients from the exercise group, of which one was possibly related vs 5 in 3 patients in the control group - In exercise group compared to control: • Reduced hospitalizations (RR = 0.97, 95%CI = 0.27;3.43) • Improved RDI (β = 7.2%, 95%CI = −5.5;20%) • Improved HRQoL (FACT-G: β = 8.6, 95%CI = 0.8;16.5; FACT-C: β = 8.0, 95%CI = 0;16) • Reduced distress (HADS: −2.1, 95%CI = −4.0;−0.2) • Improved aerobic fitness (peakVO_2_ in ml/kg/min: 2.7, 95%CI = 0.5;4.8) • Stair climbing in W = 34.4 (95%CI = 0.3;68.5) • Body composition: no significant difference
Zimmer [46]	30 mCRC during palliative treatment	Two-arm RCT	Supervised resistance and aerobic exercise intervention 60 min twice a week for 8 weeks	Mean proportion of sessions attended: 88.3%	Primary: CIPN (TOI, (FACT-NTX) Secondary; balance (GGT); muscle strength (1RM); aerobic capacity (6MWT)	- Significant differences between groups in CIPN (W8 *p* = 0.028, W12 *p* = 0.031) and neurotoxicity (W8 *p* = 0.002, W12 *p* = 0.015) in favor of the exercise group - Significant difference in balance (W8 *p* = 0.025, W12 *p* = 0.025), and muscle strength (bench press: W8 *p* = 0.014, W12 *p* = 0.014; leg press: W8 *p* = 0.001, W12 *p* = 0.011; lateral pulldown W8 *p* = 0.022, W12 *p* = 0.031) in favor of the exercise group - No significant difference in aerobic capacity

Abbreviations: AET = aerobic exercise training; AM-PAC = activity measure for post-acute care; APCT = abdominopelvic computed tomography; CIPN = chemotherapy-induced peripheral neuropathy; CRP = c-reactive protein; EORTC QLQ-C30 = European organization for research and treatment of cancer quality of life core questionnaire; ExRDI = exercise relative dose intensity, representing the percentage of exercise dose intensity during the performed sessions relative to the planned intensity and sessions; FACIT = functional assessment of chronic illness therapy; FACT-G/F/C patient reported Functional Assessment of Cancer Therapy-General/Fatigue/Colorectal; GGT = Gleichgewichtstest (German word for balance test); GI = gastrointestinal; GOG-NTX = Gynecologic Oncology Group Neurotoxicity; Hb = hemoglobin; HADS = hospital anxiety and depression scale; (HR)QoL = (health-related) quality of life; HIIT = high intensity interval training; HRR = heart rate reserve; mCRC = metastatic colorectal cancer; NRS = numeric rating scale; OS = overall survival; PFS = piper fatigue scale; PRO-CTCAE = patient-reported outcome version of the Common Terminology Criteria of Adverse Events; PWC130 = Physical Working Capacity at pulse rate 130/min; RCT = randomized controlled trial; RDI = relative dose-intensity of chemotherapy treatment; RET = resistance exercise training; RM = repetition maximum; RPE = Borg’s rate of perceived exertion; SAE = serious adverse events; SMI = skeletal muscle index; TLC = total leucocytes count; TOI = Trial Outcome Index, which is the combined score of the physical well-being, functional well-being and neurotoxicity subscales of the FACT; 6MWT = six-minute walk test

## Potential role of exercise to counteract toxicity and treatment modifications

**Fig. 2:**
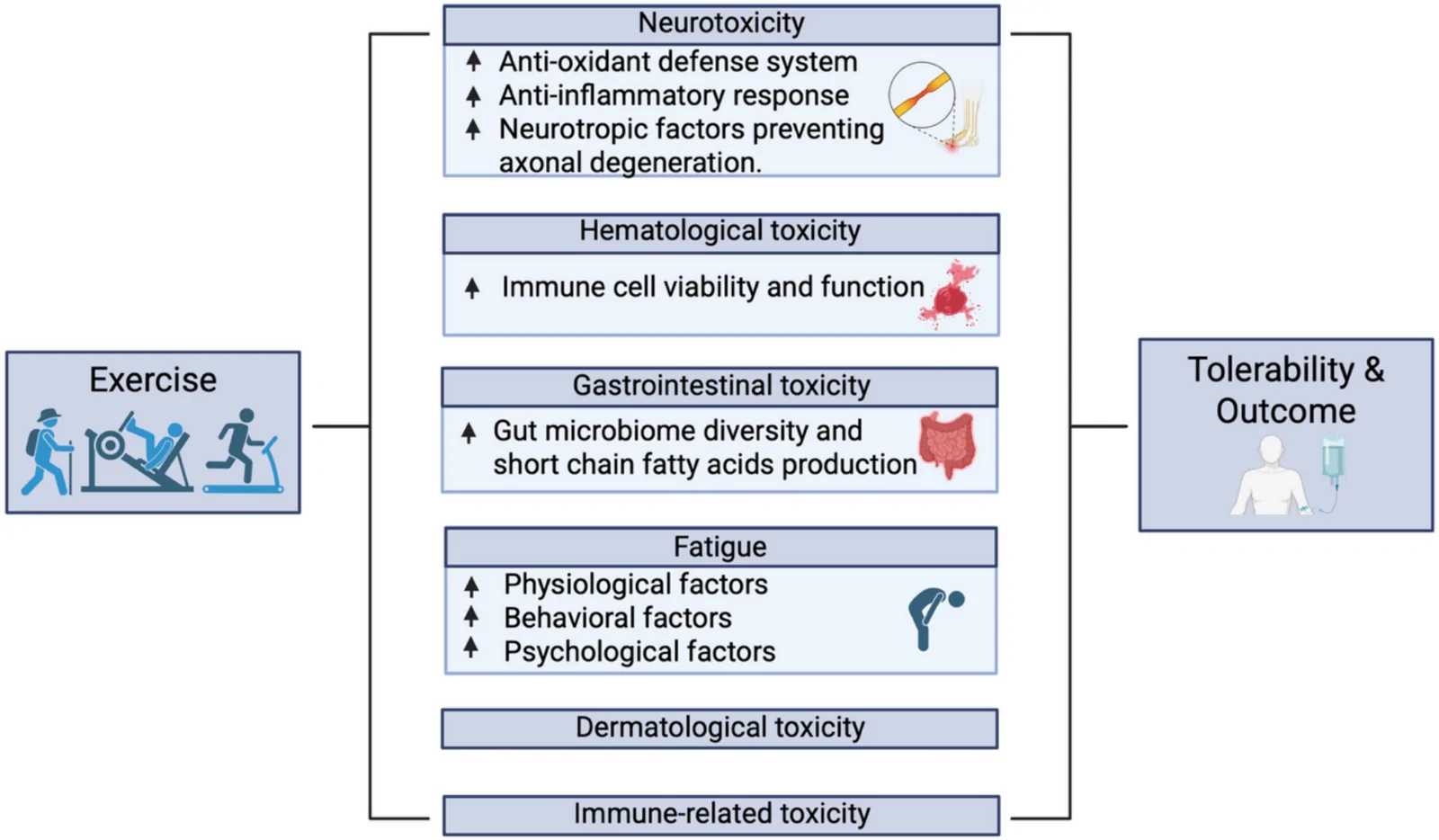
Potential mechanisms via which exercise can reduce toxicity and thereby increase treatment tolerability and outcome, Created with BioRender.com

Pharmaceutical interventions have an important role in preventing or managing toxicities like nausea, vomiting, and diarrhea. However, they do not always provide sufficient benefits and may be associated with other negative side effects, including fatigue and constipation. Exercise is a non-pharmaceutical intervention that has the potential to limit toxicity and toxicity-induced treatment modifications [48, 49] and hospitalizations [50]. A meta-analysis showed that exercise interventions effectively counteracted cancer-related fatigue during and after primary treatment, while pharmaceutical interventions did not [51]. Because of the broad range of physiological responses, exercise has the potential to counteract common CRC treatment-related toxicities, including neurotoxicity, hematological toxicity, gastrointestinal toxicity, and fatigue. The potential mechanisms of exercise in reducing these toxicities are explained below and summarized in Fig. 2.

### Neurotoxicity

Neurotoxicity is a severe side effect of platinum-based compounds including oxaliplatin. It can be divided into acute and chronic neuropathy. Acute oxaliplatin-induced peripheral neuropathy is characterized by cold-sensitive peripheral paresthesia in limbs, mouth or throat, shortness of breath and swallowing difficulties, which may appear a few hours after administration and disappear within a few days [52]. Chronic oxaliplatin-induced peripheral neuropathy does not resolve between subsequent cycles and is primarily sensory, including numbness, tingling and pain, mainly in the hands and/or feet [53]. This may progress to functional problems when continuing treatment with oxaliplatin [54]. Over half of patients with mCRC treated with oxaliplatin experience neuropathic symptoms [5, 9]. It is a leading cause of oxaliplatin dose modification and reduces QoL [53].

The mechanisms through which peripheral neuropathy occurs differ between the acute and chronic forms. Dysfunction of ion channels in membranes of neurons leading to hyperexcitability is hypothesized to play a role in the development of acute oxaliplatin-induced peripheral neuropathy, while the chronic form results from nuclear DNA damage, oxidative stress, glia activation, or neuroinflammation of peripheral neurons [52, 54]. Oxaliplatin interferes with tumor cell proliferation by forming DNA-platinum adducts, blocking DNA replication and leading to cancer cell destruction. Beside this anti-tumor activity, the platinum compounds accumulate in the dorsal root ganglion leading to apoptosis of the sensory neurons [54]. The binding of oxaliplatin to mitochondrial DNA forms adducts that cannot be repaired due to the lack of DNA repair systems within the mitochondria. Consequently, these adducts impair mitochondrial function, which enhances the production of reactive oxygen species (ROS) and exacerbate oxidative stress [54] resulting in demyelination and disruption of the cytoskeleton of peripheral nerves. The pro-inflammatory processes act as sensitizers for nociceptors contributing to the development and progression of oxaliplatin-induced peripheral neuropathy.

Previous studies among patients with cancer types other than (m)CRC reported that regular exercise may improve antioxidant capacity as indicated by enhanced activity of antioxidant enzymes (e.g., glutathione peroxidase) and reduced levels of biomarkers of oxidative damage (e.g., 8-hydroxy-2'-deoxyguanosine) [55].

Additionally, exercise may have a direct anti-inflammatory effect. During exercise, skeletal muscles produce myokines, including IL-6, IL-1ra, and IL-10, that are released into the circulation eliciting anti-inflammatory effects by stimulating the release of other anti-inflammatory cytokines and suppressing TNF-alpha [56]. This may attenuate exacerbation of oxidative stress and subsequent peripheral nerve damage. Studies among patients with cancer other than mCRC showed that exercise interventions may also enhance neurotrophic factors in serum, specifically the glial cell line-derived growth factor, brain-derived neurotrophic factor, and insulin-like growth factor 1, and help prevent axonal degeneration [57] and thereby neuropathy.

Multiple reviews of RCTs have been performed evaluating the effect of exercise interventions on the prevention or treatment of CIPN, showing promising effects in different cancer populations [58]. An umbrella review of 8 reviews, including 5 meta-analyses, showed that multimodal exercise training with at least two modalities including aerobic, resistance, balance, and/or sensorimotor training had beneficial effects on CIPN symptoms [59]. Additionally, a recent network meta-analysis, including 14 RCTs (with at least 4 including (m)CRC patients) that examined exercise effects in patients with CIPN symptoms, revealed that a combination of strengthening, stretching, and balance exercises was most effective for reducing CIPN-related pain, and that walking exercise was most effective for CIPN symptoms [60]. Most individual RCTs included in the reviews were limited by sample sizes. A recent RCT among 158 patients with lymphoma or CRC receiving treatment with vinca-alkaloids or oxaliplatin-based treatment showed that sensorimotor training or whole-body vibration resulted in a lower proportion of patients developing CIPN [61]. Also, other RCTs with sample sizes > 100 patients showed beneficial effects of exercise interventions on self-reported CIPN symptoms in women receiving chemotherapy for ovarian cancer [62] or mixed cancer populations [63, 64]. Only one small RCT (n = 30) focused specifically on patients with mCRC receiving palliative chemotherapy, of whom 17% received oxaliplatin during the trial and 63% already received oxaliplatin prior to study participation [46]. Results indicated that a multimodal exercise intervention, including balance coordination, aerobic, and resistance exercises, limits neuropathic symptoms and improves strength and balance compared with the control group. Additionally, a pilot trial among patients receiving adjuvant chemotherapy for CRC (*n* = 39, of whom 34 received oxaliplatin) reported a significantly lower proportion of patients experiencing grade 3 or 4 neurotoxicity symptoms in the exercise intervention group compared to the control group [22]. These results in patients with mCRC hold promise but should be interpreted with caution due to small sample sizes.

### Hematological toxicity

Common hematological toxicities in patients with mCRC receiving systemic treatments include anemia, leukopenia (including neutropenia), and thrombocytopenia [9]. Neutrophils are particularly vulnerable to treatment-induced bone marrow suppression. Consequently, neutropenia, including febrile neutropenia, is among the most common toxicities causing treatment modification, hospitalization, and mortality. Grade 3–4 neutropenia occurs in approximately 20–50% of patients with mCRC receiving myelosuppressive chemotherapy [5].

Neutrophils account for the majority (50–70%) of leukocytes in the circulation [65]. They are important effector cells in the innate immune system. Exercise interventions can have beneficial effects on the innate immune system in patients with cancer as it can increase natural killer (NK) cell cytotoxic activity, lymphocyte proliferation, and the number of granulocytes [66]. Results from a pilot trial among patients with breast or colon cancer showed that a 9-week aerobic and resistance exercise intervention during adjuvant chemotherapy may preserve NK cell degranulation and cytotoxicity, while it was reduced in the usual care control group [67]. Additionally, exercise has the potential to improve the number and function of neutrophils [68]. Studies in non-cancer populations have shown that moderate-intensity exercise training improves neutrophil function, including phagocytosis and oxidative burst activity, whereas very strenuous exercise training may negatively impact neutrophil function [69]. A study among 16 breast cancer survivors showed that a 16-week exercise program partially restored the chemotherapy-induced reductions in phagocytosis, compared to healthy matched controls [70]. Moreover, exercise interventions have resulted in a 10% lower hospitalization rate due to febrile neutropenia, reduced neutropenia duration, and reduced granulocyte colony-stimulating factor administration [68].

Likewise, exercise interventions during chemotherapy for patients with other types of cancer than mCRC have shown to prevent thrombocytopenia or reduce the duration of thrombocytopenia [50, 71]. In patients with mCRC, the incidence of grade 3 or 4 thrombocytopenia or anemia with current treatment regimens is low (approximately 2%) as compared to neutropenia [5].

Despite some encouraging findings that exercise interventions can limit hematological toxicity, evidence is inconsistent [68, 72]. A better understanding of which exercise type and dosage are most beneficial to optimally prevent treatment-related hematological toxicity is needed [68, 73].

### Gastrointestinal toxicity

Gastrointestinal toxicity includes mucositis, diarrhea, nausea, vomiting, and abdominal pain, and occurs frequently during fluoropyrimidine-, platinum- and irinotecan-based chemotherapy [74]. Up to 70% of patients with mCRC receiving fluoropyrimidines experience diarrhea (any grade) [5] and grade 3 or 4 toxicity occurs in up to 20%.

Irinotecan can result in early-onset and late-onset diarrhea. Early-onset diarrhea occurs during or within several hours following administration and is caused by an acute cholinergic reaction which can largely be prevented by the administration of atropine. Median time to occurrence of late-onset diarrhea is approximately 6–11 days depending on the dose and treatment schedule. Severe diarrhea can lead to treatment modification and hospitalization [74].

The irinotecan active metabolite SN-38 inhibits the enzyme topoisomerase-1 that is responsible for unfolding DNA during transcription and replication [75]. Accumulation of SN-38 damages the intestinal mucosa, resulting in disturbed fluid absorption and secretion, and consequently diarrhea [75]. Patients who are homozygous or compound heterozygous carriers of gene variants leading to UGT1A1 deficiency poorly metabolize SN-38 and are therefore at increased risk of severe toxicities, including diarrhea and neutropenia [76].

Additionally, epithelial apoptosis, induction of prostaglandins and pro-inflammatory cytokines probably contribute to diarrhea. Studies in rats showed that both irinotecan and 5-FU result in changes to the intestinal flora and mucin secretion which can contribute to the development of mucositis and diarrhea [77]. Also targeted agents such as anti-EGFR can cause diarrhea, with up to one quarter of patients experiencing grade 3 or higher [78]. Apoptosis of enterocytes, and increased inflammation are suggested to play a role [78].

In recent years, mounting evidence suggests that the gut microbiome is a key player in managing gastrointestinal toxicity [79]. Several studies showed that chemotherapy treatment reduced the richness (number of different unique taxa) and diversity (richness including taxa abundance) of the microbial community, which has in turn been associated with gastrointestinal toxicity [80, 81]. For example, the alpha diversity (within sample diversity of species) was significantly lower after chemotherapy compared to before chemotherapy [80]. Although different cytotoxic agents may differentially affect specific microbes, the microbiota generally shift towards a pathogenic gram-negative enterotype, associated with disease states including inflammatory conditions, accompanied by a significant decrease in overall species diversity [17, 80]. The gut microbiota can modulate chemotherapy toxicity and efficacy via translocation, immunomodulation, metabolism, enzymatic degradation and reduced diversity [82]. A study among patients with stage III CRC showed that those who developed diarrhea during CAPOX had a lower gut microbial community richness and community diversity compared to patients who did not experience diarrhea during treatment [81]. The modulating role of the gut microbiota in gastrointestinal toxicity is further supported by studies indicating that probiotic interventions aiming to increase community richness and diversity in patients with cancer may reduce gastrointestinal symptoms, including diarrhea [83, 84].

The gut microbiome is shaped by immanent factors such as genetics, sex, and birth delivery method, but changes over time due to breastfeeding, antibiotics or other medication use, environmental factors, diet, and exercise. Besides diet, exercise has been identified as most influential in shaping the gut microbiome [85]. Physically active people had higher microbial diversity and increased levels of butyrate-producing bacteria compared to sedentary controls, as shown by cross-sectional studies among the general population [86]. Short-chain fatty acids, such as butyrate, are produced by bacteria through the fermentation of dietary fiber, which play important roles in supporting the intestinal barrier, reducing inflammation, and regulating the immune system in the gut. Additionally, exercise interventions can alter the composition and functional capacity of the microbial community, increase the community richness, and improve resilience to microbial dysbiosis [17, 86]. However, exercise effects may vary by body mass index (BMI), with larger effects in people with a healthier BMI [87]. A recent study in 179 patients with CRC showed significantly higher microbial diversity among patients who met the exercise guidelines of at least 150 min of moderate to vigorous activity per week compared to physically inactive patients (p = 0.02 for Shannon, and p = 0.03 for Simpson diversity) [88]. Thus, gastrointestinal toxicity may be counteracted by exercise due to beneficial effects on gut microbiota. Yet, specific exercise prescriptions are needed as some gastrointestinal problems, like diarrhea, may be exacerbated by high-volume or high-intensity aerobic exercise [50].

Exercise interventions can impact the gut microbiota via several mechanisms [86]. First, exercise can modify gene expression in intraepithelial lymphocytes, downregulating pro-inflammatory cytokines and upregulating anti-inflammatory cytokines and antioxidant enzymes [89]. Second, exercise raises core body temperature and redirects blood flow from the gut, temporarily increasing gut permeability. The activation of heat-shock proteins through exercise preserves gut barrier integrity, preventing leakage of microbes and its associated gastrointestinal symptoms [86]. Third, exercise boosts secretion of bile acids into the gut, exerting antimicrobial properties. Fourth, exercise alters bowel motility, reducing transit time in the large intestine and accelerating the movement of gas through the gastrointestinal tract [90]. These effects collectively enhance gut barrier integrity, reduce chronic low-intensity inflammation, and potentially promote a healthier gut microbiota with increased diversity and butyrate-producing bacteria [91–93].

### Fatigue

Up to 10% of patients receiving systemic treatment for mCRC experience grade 3–4 fatigue, depending on the type and duration of treatment [94]. However, grade 1–2 fatigue has been reported in 57% of patients with mCRC during palliative treatment [9]. Cancer-related fatigue has been recognized as the most common persistent and distressing symptom limiting daily functioning and QoL. The etiology of fatigue associated with cancer treatment is not fully elucidated. Various biological, physiological, and psychological processes play a role in the development of fatigue, including anemia, inflammation, a dysregulation of the hypothalamic–pituitary–adrenal axis, altered energy metabolism, symptoms of anxiety and depression, sleep disturbances, and reduced physical fitness [95]. Exercise may have positive effects on all these processes, as it may increase physical fitness, reduce anemia, have an anti-inflammatory effect, and reduce anxiety, depression, and sleep disturbances [13, 56]. There is strong evidence from RCTs that exercise can limit fatigue during systemic treatment, and that the effect is larger compared with pharmacological treatments [51]. Combined aerobic and resistance exercise may have the highest probability of reducing fatigue in patients with CRC [96]. However, due to the scarcity of information on patients with mCRC, the beneficial effect of exercise on fatigue and the optimal exercise prescriptions for this population have yet to be established.

### Dermatological toxicity

Common dermatological toxicities in patients with mCRC include hand-foot syndrome and skin rash, leading to treatment modifications and reduced QoL [97]. Fluoropyrimidines, mainly capecitabine, can cause hand-foot syndrome, which is characterized by painful redness and swelling in palms and soles. Capecitabine is often replaced by S1 in case of severe hand-foot syndrome, even with dose reductions [97]. Anti-EGFR causes skin rash with associated symptoms (e.g., pain, itching) in approximately 90% of patients [98].

Symptom-limiting treatment for dermatological toxicity includes general preventive measures for rash, adequate skin care, sun protection, topical or oral steroids, and antibiotics [98]. To date, the exercise effects on skin health are generally unknown, but some evidence exists that it may decrease the thinning of the stratum corneum, limit ageing of the skin, and may help protect the skin via its anti-inflammatory and antioxidative effects [99]. The effect of exercise on dermatological toxicities is currently unknown.

### Immune-related toxicity

Immune checkpoint inhibitors can prevent immunosuppression by blocking immune checkpoints (e.g., anti-PD1/PDL1/CTLA4), thereby restoring the immune system’s antitumor response. However, the inhibition of immune checkpoints may also lead to loss of immunotolerance in lymph nodes and subsequently unleash the immune system on non-tumor cells, leading to unintended tissue damage, which manifests as severe auto-immune toxicities, such as hepatitis, colitis, hypophysitis, thyroiditis, and pneumonitis. These complications can often be mitigated by timely identification and treatment with (high dose) steroids and discontinuation of the immune checkpoint inhibitors [100].

Several pre-clinical studies in mice have investigated the effects of exercise during treatment with immune checkpoint inhibitors, showing promising results for treatment efficacy [101]. Little is known about the effects of exercise in patients receiving immune checkpoint inhibitors. Few pilot trials have shown that exercise or multidisciplinary supportive care interventions that included exercise are safe and feasible among patients with melanoma receiving immune checkpoint inhibitors [102], but effects on treatment tolerance and efficacy are yet to be determined. Notably, several clinical trials in patients with cancers other than mCRC treated with checkpoint inhibitors are currently ongoing [101].

## Concluding remarks

Survival of patients diagnosed with mCRC has improved due to multidisciplinary treatment and new or targeted systemic treatment combinations. Maintaining physical fitness throughout cancer treatment is important to endure multiple lines of systemic treatment, to maintain functional independence, the ability to perform daily life activities, and QoL. Exercise is a powerful strategy to maintain physical fitness during systemic treatments [13]. This review highlights the potential of exercise to help reduce or even prevent toxicity-induced treatment modifications. Improved treatment tolerability and maintaining a high relative dose intensity of systemic treatment can benefit survival. This review showed that results from several observational studies support the association between higher levels of physical activity, fitness, and function and mCRC treatment toxicity and outcome. However, observational studies limit the ability to make causal inferences.

In the era of evidence-based medicine, RCTs are considered the gold standard for ascertaining treatment efficacy. Several RCTs showed that exercise during systemic treatment for mCRC is feasible and safe, with no evidence of exacerbation of symptoms or treatment-related side effects. Currently, one RCT examining the effects of exercise on toxicity-induced treatment modification in patients with mCRC receiving systemic treatment is ongoing (NCT04754672), of which findings on feasibility and patient perceptions have been reported [39]. Next to knowledge on the effects of exercise on clinical outcome from RCTs, it is important to understand how these interventions achieve their effect, as this may help to identify effective intervention components and provide causal explanations. This mechanism-based medicine can further personalize interventions for the individual patient. Personalized exercise interventions include targeted exercise prescriptions in terms of frequency, intensity, type, and time to specific endpoints, taking into account treatment and personal characteristics.

This review reported several mechanistic pathways via which exercise can help prevent common dose-limiting toxicities of systemic treatment for patients with mCRC. First, exercise can help prevent neurotoxicity by enhancing the antioxidant defense system, anti-inflammatory responses, and neurotropic factors in serum preventing axonal degeneration. Second, exercise can help prevent neutropenia and other hematological toxicities by its effect on immune cell viability and function. Third, exercise may help to prevent gastrointestinal toxicity via modulating the gut microbiome, increasing community richness and diversity, thereby improving resilience to systemic treatment. Finally, exercise can help to limit fatigue via several physiological, behavioral, and psychological pathways. Although physical exercise aims to improve physical fitness, the described underlying mechanistic pathways linking physical exercise to toxicity suggest that the benefits of exercise do not necessarily need to be mediated by physical fitness. Nevertheless, maintaining physical fitness is important for physical functioning.

Future studies should be directed towards understanding the causal effects and underlying mechanisms of exercise during systemic treatment, including novel immunological and targeted agents, on treatment toxicity and outcomes in patients with mCRC in order to improve their survival and QoL. Such studies are challenged by the large heterogeneity in chemotherapy treatment tolerability, related to individual factors such as adipose tissue [103], muscle mass [11], psychological strength [18], physical fitness [12], and pharmacokinetics [104]. Future mechanistic insights are needed to identify the potential and limits of the causal effects of exercise on specific toxicities.

## Data Availability

No datasets were generated or analysed during the current study.
